# Clinical Forms of Canine Visceral Leishmaniasis in Naturally *Leishmania infantum*–Infected Dogs and Related Myelogram and Hemogram Changes

**DOI:** 10.1371/journal.pone.0082947

**Published:** 2013-12-23

**Authors:** Roney de Carvalho Nicolato, Raquel Trópia de Abreu, Bruno Mendes Roatt, Rodrigo Dian de Oliveira Aguiar-Soares, Levi Eduardo Soares Reis, Maria das Graças Carvalho, Cláudia Martins Carneiro, Rodolfo Cordeiro Giunchetti, Leoneide Erica Maduro Bouillet, Denise Silveira Lemos, Wendel Coura-Vital, Alexandre Barbosa Reis

**Affiliations:** 1 Laboratório de Imunopatologia, Núcleo de Pesquisas em Ciências Biológicas/NUPEB, Universidade Federal de Ouro Preto, Ouro Preto, Minas Gerais, Brazil; 2 Laboratório de Pesquisas Clinicas, Departamento de Análises Clínicas, Escola de Farmácia, Universidade Federal de Ouro Preto, Ouro Preto, Minas Gerais, Brazil; 3 Laboratório de Hematologia Clínica, Faculdade de Farmácia, Universidade Federal de Minas Gerais, Belo Horizonte, Minas Gerais, Brazil; 4 Laboratório de Biologia das Interações Celulares, Departamento de Morfologia, Universidade Federal de Minas Gerais, Belo Horizonte, Minas Gerais, Brazil; 5 Laboratório de Imunologia e Genômica de Parasitos – Departamento de Parasitologia, Universidade Federal de Minas Gerais, Belo Horizonte, Minas Gerais, Brazil; 6 Laboratório de Epidemiologia das Doenças Infecto Parasitárias - Pós-graduação em Infectologia e Medicina Tropical, Universidade Federal de Minas Gerais, Belo Horizonte, Brazil; Instituto de Higiene e Medicina Tropical, Portugal

## Abstract

Hematological analysis has limited applications for disease diagnosis in *Leishmania infantum*–infected dogs, but it can be very important in evaluating the clinical forms of the disease and in understanding the evolution of canine visceral leishmaniasis (CVL) pathogenesis. Recently, we demonstrated that alterations in leucopoiesis and erythropoiesis are related to clinical status and bone marrow parasite density in dogs naturally infected by *L. infantum*. To further characterize these alterations, we evaluated the association between the hematological parameters in bone marrow and peripheral blood alterations in groups of *L. infantum*–infected dogs: asymptomatic I (AD-I: serum negative/PCR+), asymptomatic II (AD-II: serum positive), oligosymptomatic (OD), and symptomatic (SD). Results were compared with those from noninfected dogs (NID). The SD group was found to present a decrease in erythropoietic lineage with concomitant reductions in erythrocytes, hemoglobin, and hematocrit parameters, resulting in anemia. The SD group also had increased neutrophils and precursors and decreased band eosinophils and eosinophils, leading to peripheral blood leucopenia. In the AD-II group, lymphocytosis occurred in both the peripheral blood and the bone marrow compartments. The SD group exhibited lymphocytosis in the bone marrow, with lymphopenia in the peripheral blood. In contrast, the AD-I group, showed no significant changes suggestive of CVL, presenting normal counts in bone marrow and peripheral blood. Our results showed for the first time that important changes in hematopoiesis and hematological parameters occur during ongoing CVL in naturally infected dogs, mainly in symptomatic disease. Taken together, our results based on myelogram and hemogram parameters enable better understanding of the pathogenesis of the anemia, lymphocytosis, and lymphopenia, as well as the leucopenia (eosinopenia and monocytopenia), that contribute to CVL prognosis.

## Introduction

In urban areas, the domestic dog is the main reservoir of *Leishmania infantum*, and canine visceral leishmaniasis (CVL) has been associated with human cases of disease [1]–[2]. Given the increased incidence of CVL in the last decade, this disease has become highly epidemiological relevant, and the intense cutaneous parasite burden that has been reported in infected dogs may add to the spread of disease [3]–[5].

Infected dogs may present with a wide range of clinical symptoms, from apparently healthy to critically diseased [6]–[7], depending on the balance between cellular and humoral immune responses [7]–[8]. Hematological parameters and the serum biochemical profile in *L. infantum*–infected dogs are of limited use for disease diagnosis. However, they can be very important biomarkers for evaluating the clinical progress of infected animals and may also contribute to the understanding of CVL pathogenesis [9]–[12].

The genesis of hematological alterations in both red and white blood cell series is often related to bone marrow disorders such as dysplasia and aplasia [13]–[14]. A detailed examination of bone marrow provides the hematopoietic status of an individual [15], thus indicating that bone marrow disorders are related to peripheral blood alterations. Moreover, when used in combination with a complete blood count, examination of bone marrow smears provides information about the hematopoietic system that might otherwise be missed by analysis of the peripheral blood alone [16].

Until now, the myelopoietic alterations in dogs naturally infected by *L. infantum* were unclear. Through this investigation, we show that bone marrow evaluation provides a useful method for elaborating a prognosis for CVL as well as contributes with the diagnosis of cases with a strong suspicion of CVL that cannot be confirmed by serological tests [11]. Tropia de Abreu et al. [11] previously showed the impact of clinical status and distinct bone marrow parasite density on hematopoietic activity during CVL in seropositive animals. Foglia Manzillo et al., [17] have shown that any susceptible young dogs exposed to different transmission incidences have similar chances to develop an active infection and become symptomatic. In recent years in a cross sectional study our group has sought biomarkers of resistance and susceptibility, especially in asymptomatic dogs that may be infected (as determined by PCR) but not detected by serological methods. There is a high prevalence of such dogs in urban areas [2], and although their infectious status is not detected in conventional serology, they are more likely to seroconvert [4]. These animals appear to have an immune response profile similar to that of uninfected dogs, but their cellular response shows increased monocytes (CD14^+^) and T lymphocytes, particularly CD4^+^ T subpopulation, compared with symptomatic dogs [18]. Herein, we report a detailed analysis of the changes observed in the bone marrow cellular profile and the consequences of these changes in peripheral blood cells in dogs naturally infected by *L. infantum*. Our analysis involved dogs with distinct clinical statuses, including those that were asymptomatic seronegative/PCR+. In addition, we focused on the association between myelogram and hemogram changes that may offer an additional prognostic tool to understand the clinical progression of CVL.

## Materials and Methods

### Selection of Dogs and Experimental Design

Forty male and female mongrel dogs aged 2 to 6 years old from the endemic area of Belo Horizonte, Minas Gerais, Brazil, were selected on the basis of serological tests for *Leishmania* spp. (immunofluorescence antibody test [IFAT] and enzyme-linked immunosorbent assay [ELISA], Biomanguinhos/Fiocruz). Seven dogs with ELISA and IFAT titers <1/40 and negative PCR results were considered to be noninfected dogs (NID), and 27 dogs with ELISA and IFAT titers ≥1/40 were considered to be seropositive and infected by *Leishmania* spp. Six dogs with negative ELISA results, IFAT titer <40, and positive PCR results were considered infected. Positive infection was confirmed in buffy coat samples by PCR [19], and the species of *Leishmania* responsible was determined by restriction fragment length polymorphism–PCR [20] and to confirm the non-infection status in control dogs we also performed PCR in bone marrow biopsies and all dogs of this group were negative. The study was conducted from June 2008 to August 2009 after approval by the ethics committees for the use of experimental animals of the Federal University of Ouro Preto (CETEA/UFOP 032/2007), Federal University of Minas Gerais (CETEA/UFMG 020/2007), and the Municipal Health Secretariat of Belo Horizonte City Council, Minas Gerais State, Brazil (CEP-SMSA/PBH 001/2008). Owners of the dogs participating in the project were informed of the research objectives and were required to sign the Informed Consent Form before sample.

### Clinical Evaluations

Twenty-seven mongrel dogs that were serum and PCR positive for *Leishmania* infection were clinically classified according Coura-Vital et al. [18] and divided into three distinct categories based on external clinical signs. Dogs with no clinical signs were classified as asymptomatic dogs II (AD-II, n = 7); dogs with three or fewer clinical signs, including opaque bristles, localized alopecia, and/or moderate weight loss, were classified as oligosymptomatic (OD, n = 8); and dogs with characteristic clinical signs of CVL such as opaque bristles, severe weight loss, onychogryphosis, cutaneous lesions, apathy, and keratoconjunctivitis were classified as symptomatic dogs (SD, n = 12). Seronegative dogs without clinical signs that had positive molecular results for *L. infantum* were classified as asymptomatic dogs I (AD-I, n = 6). Dogs with no clinical signs and negative serological and molecular results composed the noninfected group (NID, n = 7).

### Blood Sample Collection

Peripheral blood (5 mL) from the brachiocephalic vein was collected into tubes containing ethylene diamine tetraacetic acid (EDTA) at a final concentration of 1 mg/mL. Erythrocytes and leucocytes were quantified using an automatic cell counter (Model 2800 Vet, Mindray). Differential leucocyte counts were performed by examination of at least 200 leucocytes in Giemsa-stained blood smears by light microscopy.

### Bone Marrow Aspiration and Myelogram

Animals were sedated with an intravenous dose (8 mg/kg body weight) of sodium thiopental (Thionembutal®; Abbott Laboratories, São Paulo, Brazil), and bone marrow fluid was removed from the ventral region of the sternum or from the iliac crest. Slide smears of the bone marrow aspirates were Giemsa-stained and examined under an optical microscope. Leucopoietic and erythropoietic alterations were evaluated by differential counting of 500 cells with reference to the cellular classifications of Penny [21] and Jain [22]. For cytological evaluation, slide smears were examined under an Olympus Optical Co. (Tokyo, Japan, model CH3RF100 optical microscope). Images were captured at ×100 magnification using a Leica DM5000B micro-camera (Leica Microsystems-Switzerland Ltd., Heerbrugg, Switzerland) and Leica Application Suite software (version 2.4.0 R1). Twenty random images (total area = 1.5×10^6^ µm^2^) were determined to be sufficient for the representative analysis of a slide.

### Statistical Analysis

Statistical analysis was performed using GraphPad Prism 5.0 (Prism Software, Irvine, CA, USA). The normality of the data was assessed using the Kolmogorov–Smirnoff test. Considering the nonparametric nature of all data sets, Kruskal–Wallis tests were used to investigate differences between the four groups, followed by Dunn’s test for pairwise comparisons. Spearman’s rank correlation was computed to investigate associations among myelogram and peripheral blood parameters as well as among cell counts and clinical groups. In all cases, the differences were considered significant when the probabilities of equality, p-values, were ≤0.05.

## Results

### Marked Anemia, Eosinopenia, Lymphopenia, and Monocytopenia are Hallmarks of Hematological Dysfunction Associated with Severe Clinical Presentation of CVL

Evaluation of the hematological parameters revealed severe anemia in the SD group, with significantly lower numbers for erythrocytes, hemoglobin, and hematocrit compared with the NID, AD-I, and AD-II groups (p<0.05; Table 1). In the white blood cell analysis, significantly lower absolute counts of eosinophils were observed in the AD-II, OD, and SD groups compared with the NID group. Interestingly, significantly higher absolute counts of lymphocytes were observed in the AD-II group compared with the NID and SD groups. Furthermore, absolute counts of monocytes were significantly lower in the SD group compared with the NID group (Table 1).

**Table 1:** Assessment hematological parameters in natural *L. infantum*-infected and non-infected dogs.

Hematological parameters	Clinical groups				
	NID	AD-I	AD-II	OD	SD
Erythrocytes (10^6^/mm^3^)	6.8 (6.6–7.0)	7.3 (6.6–7.5)	6.2 (6.0–6.3)	5.7 (5.1–6.1)	**4.1 (3.4–5.0)^a,b,c^**
Hemoglobin (g%)	16.9 (16.8–17.1)	15 (13.0–16.2)	15.4 (14.5–15.9)	13.8 (13.6–16.4)	**10.5 (7.7–10.9)^a^**
Hematocrit (%)	49.3 (48.5–49.9)	46.1 (40.5–49.4)	42.1 (42.0–44.2)	39.9 (37.6–44.7)	**29.3 (22.6–33.4)^a^**
Leucocytes (10^3^/mm^3^)	11.4 (11.1–16.4)	13.3 (9.3–14.1)	13.8 (9.3–16.2)	12.6 (10.6–14.6)	11.7 (7.5–13.0)
Granulocytes	8.7 (7.3–12.6)	8.1 (5.3–11.0)	5.2 (4.5–9.1)	7.7 (6.6–11.6)	8.8 (6.6–11.5)
Total neutrophils	7.4 (5.2–10.6)	5.7 (3.7–9.6)	4.9 (4.3–7.4)	7.4 (5.8–10.4)	8.7 (6.5–9.7)
Band neutrophils	0.8 (0.5–1.0)	0.4 (0.3–0.8)	0.7 (0.3–0.9)	1.0 (0.6–1.4)	1.4 (0.8–1.7)
Segmented neutrophils	6.3 (4.9–9.5)	5.2 (2.9–9.0)	3.9 (3.7–6.6)	6.6 (5.0–8.7)	7.2 (5.2–8.2)
Eosinophils	2.2 (1.5–2.5)	1.4 (1.1–2.1)	**0.4 (0.2–1.0)^a^**	**0.7 (0.4–1.2)^a^**	**0.2 (0.1–0.8)^a^**
Lymphocytes	2.4 (1.4–2.7)	2.4 (1.9–4.0)	**3.8 (3.3–5.5)^a^**	3.2 (2.2–3.9)	**1.4 (0.8–2.1)^c^**
Monocytes	1.3 (1.0–1.6)	1.0 (0.6–1.5)	1.0 (0.6–1.4)	0.6 (0.5–1.1)	**0.5 (0.4–0.7)^a^**
Platelets (10^5^/mm^3^)	1.9 (1.3–2.7)	2.3 (2.0–2.7)	1.9 (1.4–2.1)	2.0 (1.9–2.1)	1.7 (1.4–2.2)

Results are shown as the median and interquartile range. NID, noninfected dogs; AD-I, asymptomatic dogs I; AD-II, asymptomatic dogs II; SD, symptomatic dogs. Letters *a*, *b* and *c* indicate statistically significant differences (p<0.05) compared to NID, AD-I, and AD-II, respectively.

### Hypoplasia of the Erythroblastic Series was the Main Alteration in Myelograms of Symptomatic Dogs during Ongoing CVL

**Figure 1:**
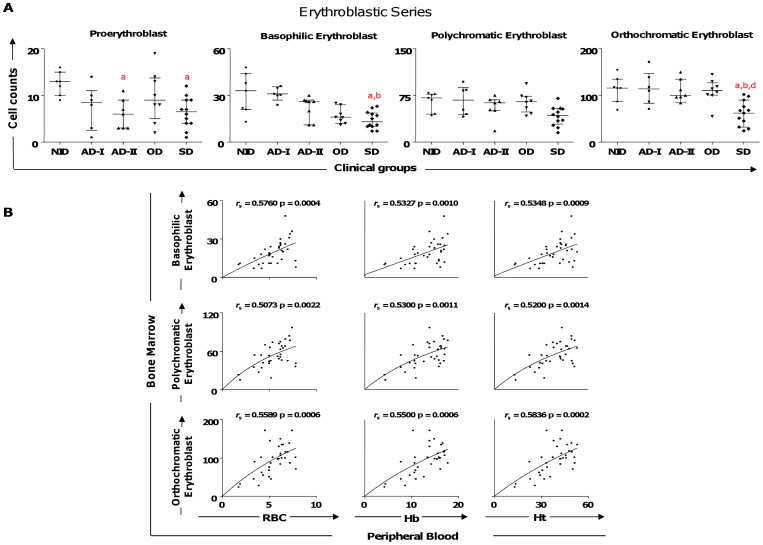
Profiles of the erythroblastic cell series in dogs naturally infected by *Leishmania infantum*. (A) Animals categorized according to their clinical status into asymptomatic I (AD-I, n = 6), asymptomatic II (AD-II, n = 7), oligosymptomatic (OD, n = 8), symptomatic (SD, n = 12), or noninfected (NID, n = 7) groups. Significant differences at p<0.05 are identified by the letters a, b, and d in comparison to NID, AD-I, and OD, respectively. The results are expressed on graphs as scattering of individual values and are also shown as median and interquartile range values. (B) Correlation between erythroblastic series cells and hematological parameters (erythrocytes [RBC], hemoglobin [Hb], and hematocrit [Ht]) considering all groups of dogs infected by *L. infantum*. The results are expressed as scattering of individual values. Spearman’s rank correlation (*r*_s_ and p values) are shown in the graphs.

Evaluation of bone marrow parameters related to the red cell series showed fewer proerythroblasts in the AD-II and SD group compared to the NID group (Fig. 1A). Additionally, a lower count in the basophilic erythroblasts was observed in the SD group compared with the NID and AD-I groups. Significantly lower counts in the end stages of erythropoietic lineage (orthochromatic erythroblast) were also observed in the SD group compared with the NID, AD-I, and OD groups (Fig. 1A).

### Lower Erythroblastic Series Counts were Correlated to Lower Levels of Erythrocytes, Hemoglobin, and Hematocrit in the Erythrogram of the CVL

To evaluate if the bone marrow disturbance during CVL is reflected in alterations of peripheral blood cells, we conducted an analysis involving counts of proerythroblasts, basophilic erythroblasts, polychromatic erythroblasts, and orthochromatic erythroblasts with the hematological parameters (erythrocyte [RBC], hemoglobin [Hb], and hematocrit [Ht] obtained in an erythrogram; Fig. 1B). Our data showed a positive correlation among basophilic, polychromatic, and orthochromatic erythroblasts with peripheral blood parameters (RBC, Hb, and Ht) in all groups of *L. infantum*–infected dogs (Fig. 1B).

### Increased Neutrophilic and Decreased Eosinophilic Series are Associated with Severe Disease

**Figure 2:**
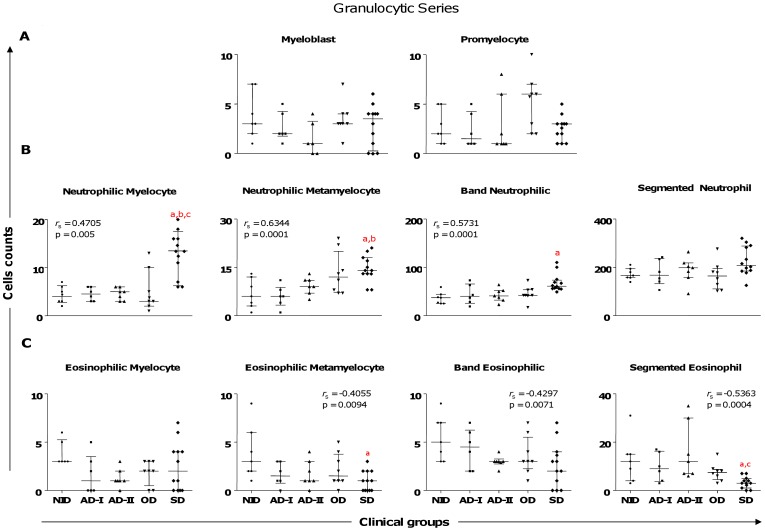
Profiles of the precursors of granulocytic lineage cells in dogs naturally infected by *Leishmania infantum* categorized according to clinical status. Animals categorized according to their clinical status into asymptomatic I (AD-I, n = 6), asymptomatic II (AD-II, n = 7), oligosymptomatic (OD, n = 8), symptomatic (SD, n = 12), or noninfected (NID, n = 7) groups. Significant differences at p<0.05 are identified by the letters a, b, and c in comparison to NID, AD-I, and AD-II respectively. The results are expressed in graphics as scattering of individual values and are also shown as median and interquartile range values. Spearman’s rank correlation (*r*_s_ and p values) are also shown.

We found several alterations in the granulocytic series in *L. infantum–*infected dogs (Fig. 2). The neutrophilic series displayed a significantly higher counts in (*i*) neutrophilic myelocytes in the SD group compared with the NID, AD-I, and AD-II groups; (*ii*) neutrophilic metamyelocytes in the SD group compared with the NID and AD-I groups; and (*iii*) band neutrophilic in the SD group compared with the NID group (Fig. 2B). Additionally, a positive correlation existed between clinical ongoing CVL and the neutrophil precursors (Fig. 2B). Moreover, in the eosinophil precursor series (Fig. 2C), significantly lower counts were observed for eosinophilic metamyelocytes in the SD group compared with the NID group; further, the SD group had lower segmented eosinophil counts compared with the NID and AD-II groups. Our data showed a nonsignificant tendency for band eosinophilic to decrease with clinical progression. A negative correlation between clinical ongoing CVL and these eosinophil precursors was also observed (Fig. 2C).

### Peripheral Blood Eosinopenia in All Seropositive Dogs was Predicted for a Decrease of Eosinophil Precursors in Bone Marrow

**Figure 3:**
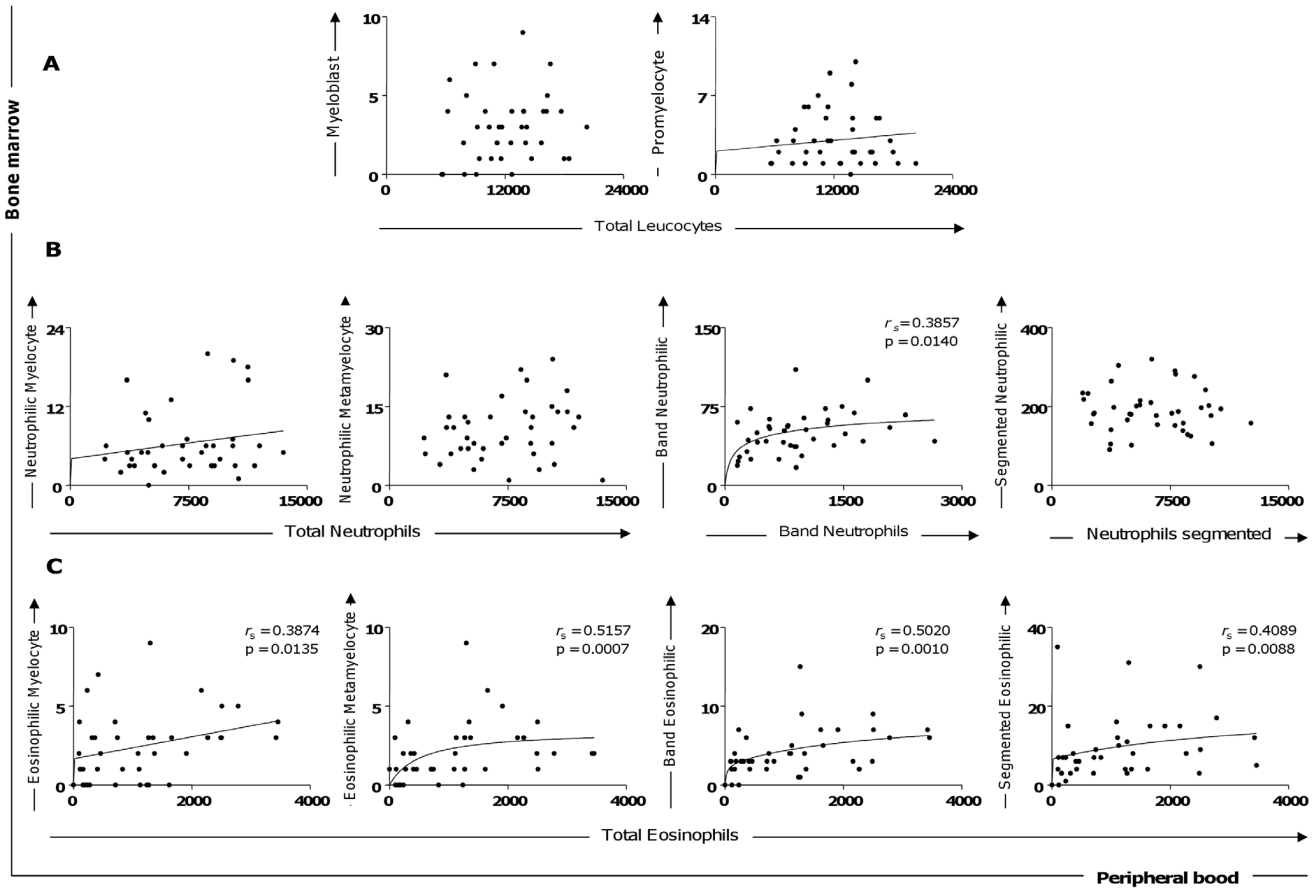
Correlation between bone marrow (precursors of granulocytic lineage cells) and peripheral blood (with cells) parameters. (A) Myeloblast versus total leucocytes and promyelocyte versus total leucocytes. (B) Neutrophilic myelocyte versus total neutrophils, neutrophilic metamyelocyte versus total neutrophils, band neutrophilic versus band neutrophils, and segmented neutrophilic versus neutrophils segmented. (C) Eosinophilic myelocytes, eosinophilic metamyelocytes, band eosinophils, and segmented eosinophils versus total eosinophils of peripheral blood. Correlation between peripheral blood and bone marrow of the granulocyte series. The results are expressed as scattering of individual values. Spearman’s rank correlation (*r*_s_ and p values) are also shown in the graphics.

In order to evaluate if the bone marrow cell alterations during CVL are reflected in changes for circulating leucocytes, we analyzed the correlation of both myeloblasts and promyelocytes with total leucocytes (Fig. 3A). Moreover, correlation analysis was performed for neutrophilic precursors and their corresponding peripheral blood leucocytes. Additionally, the correlation between eosinophilic precursors and total eosinophils in peripheral blood was evaluated. A positive correlation was detected for the number of circulating eosinophils and the number of bone marrow eosinophils (myelocyte, metamyeloyte, band, and segmented) in all analyses (Fig. 3C). Also, our data demonstrated a positive correlation between circulating band neutrophils and the band neutrophilic population in the bone marrow compartment (Fig. 3B).

### Bone Marrow Lymphocytosis in Seropositive Animals is Associated with Increased Circulating Lymphocytes in AD-II and Lymphopenia in Symptomatic CVL

**Figure 4:**
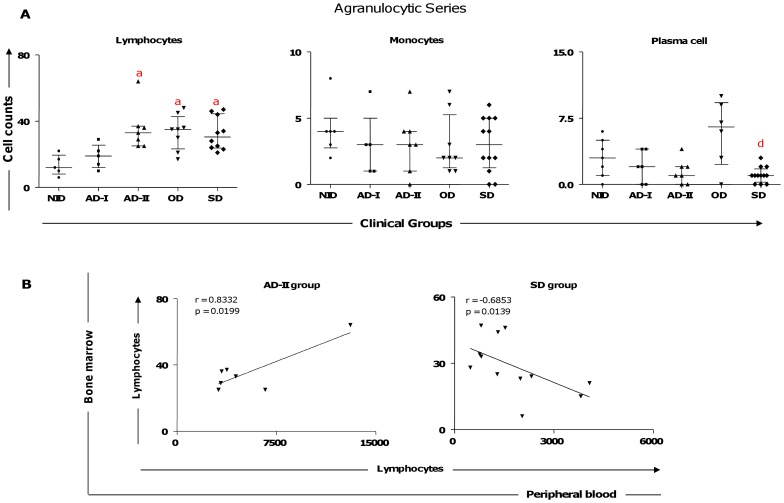
Profiles of the agranulocytic cell series in bone marrow of dogs naturally infected by *Leishmania infantum*. (A) Animals categorized according to their clinical status as asymptomatic I (AD-I, n = 6), asymptomatic II (AD-II, n = 7), oligosymptomatic (OD, n = 8), symptomatic (SD, n = 12), or noninfected (NID, n = 7) groups. Significant differences at p<0.05 are identified by the letter a and d in comparison to NID and OD, respectively. The results are expressed on graphs as scattering of individual values and are also shown as median and interquartile range values. (B) Correlation between peripheral blood and bone marrow of the lymphocytes in AD-II and SD groups. The results are expressed as scattering of individual values. Pearson correlation indexes (r and p values) are shown on the graphs.

Our bone marrow compartment data showed higher lymphocyte counts were closely related to the AD-II, OD, and SD groups compared to the NID group (Fig. 4A). In contrast, the SD group displayed lower plasma cell counts compared with the OD group (Fig. 4A). To confirm and extend our findings on immunopathological changes in lymphocytes, we performed correlation analyses between the circulating lymphocyte counts and lymphocyte counts from bone marrow (Fig. 4B). Our data showed bone marrow lymphocyte counts and peripheral blood lymphocytes were positively correlated in the AD-II group. We also detected a negative correlation for these parameters in the SD group (Fig. 4B).

### Cytological Findings such as Emperipolesis and Mott Cells were found in the Bone Marrow of Symptomatic Dogs

**Figure 5:**
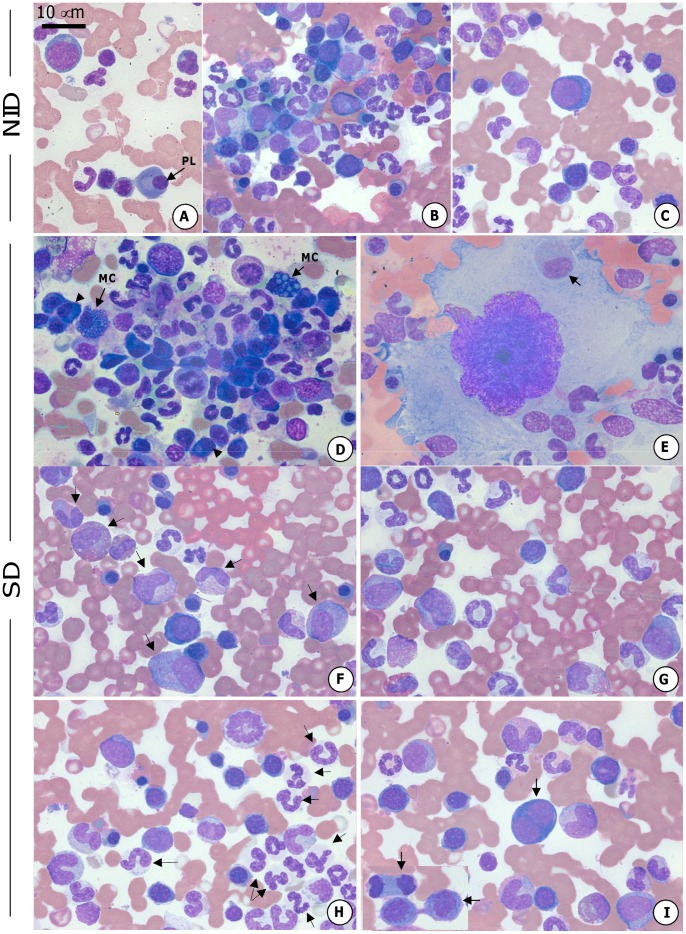
Canine bone marrow cytology. The bone marrow smears derived from the NID dogs are demonstrated (A–C). The SD group demonstrated plasma cell multiplication (arrow-head) and Mott cells (MC) (D). Emperipolesis, the penetration of hematopoietic cells into the cytoplasm of megakaryocytes (arrow) (E). Increase of precursor leucopoetic lineage cells (F) and decrease of all erythropoietic lineage cells (G) in the SD group. Symptomatic dogs presented an increase in neutrophilic lineage cells (H) and erythroblastic series cells in mitotic cell division (I) (arrow). Slides were stained with Giemsa; bar = 10 µm.

Differential cell counting in bone marrow smears revealed distinct cell morphological changes in symptomatic dogs (Fig. 5). The main cytological findings observed in the bone marrow smears derived from the SD group are presented in Fig. 5D and compared with the NID group in Fig. 5A–C. Multiple plasma cells and Mott cells could be readily observed (Fig. 5D). Additionally, we found emperipolesis, a phenomenon that occurs with the penetration of hematopoietic cells into the cytoplasm of megakaryocytes as shown in Fig. 5E. The SD group showed a higher frequency of precursors of leucopoetic lineage cells (Fig. 5F) and lower frequency of all erythropoietic lineage cells (Fig. 5G) compared to the NID group (Fig. 5B). Further analysis in symptomatic dogs showed higher amounts of neutrophilic lineage cells (Fig. 5H) and erythroblastic series cells undergoing mitotic cell division (Fig. 5I) in relation to the NID group (Fig. 5C).

## Discussion

Hematological and myelopoietic alterations have been reported in human visceral leishmaniasis and CVL; however, few studies have shown hematopoietic alterations caused by *L. infantum* in conjunction with consideration of the bone marrow compartment as a major target organ of the parasites [9]. This study evaluated the relationship between peripheral blood and leucopoietic/erythropoietic changes in association with the distinct clinical status of dogs naturally infected by *L. infantum*.

One the most remarkable characteristics of CVL-associated hematological disorders is anemia being reported as nonregenerative normocytic or normochromic [6], [18], [23]–[26]. In the current study, we documented that severe CVL is associated with a disorder in the erythroid bone marrow compartment, with significantly decreased red blood cell counts and hemoglobin and hematocrit values in symptomatic dogs, corroborating previous studies and elucidating the genesis of anemia in active CVL. It is possible that anemia could be related to factors such as formation of colonies impaired and increased hemolysis in enlarged spleen and liver associated with an inflammatory response to *L. infantum* infection [27]. In addition, a reduced plasma iron in the presence of greatly increased iron stores suggested that reticuloendothelial hyperplasia was accompanied by abnormal iron retention by macrophages typical of the anemia of chronic disorders. This may limit the erythropoietic response to anemia in chronic visceral leishmaniasis [28].

De Luna et al. [29] suggested that anemia could result from impaired erythrocyte membrane fluidity in CVL, leading to mechanical sequestration in the spleen and/or alterations in receptor–ligand erythrocyte cytoadherence mechanisms. Additionally, we described an important relation between bone marrow and peripheral blood cellularity; specifically, erythropoietic cell lineages (especially basophilic erythroblast and orthochromatic erythroblast) decreased according to the clinical progress of CVL. Amusategui et al. [30] reported that asymptomatic dogs showed higher erythrocyte counts and hemoglobin and hematocrit values compared with oligosymptomatic and symptomatic dogs. These results allow speculation that severe CVL is associated with a bone marrow dysfunction characterized by diminished erythropoiesis, due to intense bone marrow parasitism, which is a hallmark of symptomatic dogs [9].

To evaluate the patterns of leucocyte alteration in the blood and bone marrow compartment, the white blood cell series was analyzed. Lymphocytosis in the AD-II group and leucopenia in symptomatic disease, characterized by monocytopenia, lymphocytopenia, and eosinopenia, were observed. These data corroborate findings from Reis et al. [9], [26]. However, Da Costa-Val et al. [12] did not observe a correlation between leucocyte values and clinical signs in CVL, except for lymphocytes in asymptomatic dogs compared with other groups. Among the precursors of granulocytic lineage, we described a high frequency of myelocytes, methamyelocytes, and band neutrophils in the bone marrow of symptomatic dogs as well as a positive correlation with CVL evolution. These results suggest a high demand for neutrophilic cells in symptomatic dogs as a result of the inflammatory response in multiple organs affected by parasitism [7]. In addition, Amusategui et al. [28] reported that asymptomatic dogs presented a decrease in circulating neutrophils. Moreover, increased neutrophil counts in the spleens of dogs naturally infected by *L. infantum* have been described [31]. Eosinophilic hypoplasia reported in the SD group, comprising a reduction in eosinophilic metamyelocytes and segmented eosinophils, and a negative correlation with circulating eosinophils upon CVL progression could indicate bone marrow dysfunction during this clinical stage of the infection, which may have contributed to the severe eosinopenia seen in the SD group. Similar results were observed by Tryphonas et al. [32] who reported a reduction in eosinophilic precursors from bone marrow at the end stage of CVL. Further analyses revealed a positive correlation between band neutrophilic (bone marrow) and band neutrophils (peripheral blood), in addition to total eosinophils (peripheral blood) and eosinophil precursors (myelocytes, metamyelocytes, band eosinophilic, and segmented eosinophil). We believed that these alterations could be occurring due to the stimulation provided by the *L. infantum* bone marrow parasitism as previously shown by Tropia de Abreu et al. [11] in symptomatic dogs and dogs with high bone marrow parasitism. However, the role of the neutrophils and eosinophils during ongoing CVL remains unclear, and no functional analysis in these cell populations has been described.

Our data demonstrated that lymphocytosis in the bone marrow was associated with lymphocytosis in the peripheral blood as observed in the AD-II group, which confirmed the hypothesis previously described by Reis et al. [9]. However, in symptomatic dogs, lymphocytosis in bone marrow was correlated with lymphopenia in the peripheral blood. This phenomenon can be explained by lymphocytes and phagocytes migrating to the affected lymphoid organs to build the inflammatory response that typically occurs with CVL. In this context, bone marrow lymphocytosis in CVL occurs due to a compensatory response that provides lymphocytes to target organs affected by the parasite, which is reflected by the peripheral blood lymphopenia observed in advanced stages of CVL [9], [33]. Interestingly, while asymptomatic dogs achieved increased lymphocyte counts in the bone marrow compartment and in the blood, symptomatic dogs failed to retain this cell population in the blood compartment. We hypothesize that an increased parasite burden in different tissues, which is typical in symptomatic dogs [7], [34]–[35], would contribute to lymphocytes migrating from blood to the parasitized tissues, resulting in lower counts for circulating lymphocytes.

Finally, we further assessed morphological bone marrow alterations in dogs naturally infected by *L. infantum* that displayed different clinical stages of CVL. We also described emperipolesis in symptomatic dogs, which was previously reported by Foglia Manzillo et al. [36] in CVL. This phenomenon has been observed in humans [37] and in rats [38] under conditions of chronic blood loss, cancer, myeloproliferative disorders, and reactive thrombocytosis. Moreover, we found Mott cells in the bone marrow compartment of symptomatic dogs in association with plasmocytosis, which has been described as hyperactivity of plasma cells that contain immunoglobulin granules in their cytoplasm [16], [22]. Previous studies reported increased counts of plasma cells and Mott cells in CVL resulting from antigenic stimulation associated with intense parasitism in the bone marrow compartment [39].

Considering the results obtained in the present study, we can observe some biomarkers that can be used in the prognosis of CVL such as: lymphocytosis in the bone marrow, and lymphopenia in the peripheral blood in SD. In contrast, the AD-I group, showed no significant changes suggestive of CVL, presenting normal counts in bone marrow and peripheral blood. Our results showed for the first time that important changes in hematopoiesis and hematological parameters occur during ongoing CVL in naturally infected dogs, mainly in symptomatic disease. Taken together, our results based on myelogram and hemogram parameters enable better understanding of the pathogenesis of the anemia, lymphocytosis, and lymphopenia, as well as the leucopenia (eosinopenia and monocytopenia), that contribute to indentify some biomarkers for CVL prognosis.

In conclusion, our study shows that the clinical evolution of CVL in dogs naturally infected by *L. infantum* is associated with alterations in hematopoiesis and hematological parameters mainly observed in symptomatic dogs. In contrast, the AD-I group showed no significant changes in either compartment, presenting normal count values in both bone marrow and peripheral blood as observed in the NID group. The myelogram alterations were closely related to absolute cell counts in the peripheral blood, suggesting that disturbances in myelopoiesis are directly reflected in white and red circulating cells through consequences such as the anemia and leucopenia (eosinopenia, monocytopenia, and lymphopenia) typical in dogs with severe disease.
